# Metabolomics as a Potential Chemotaxonomical Tool: Application in the Genus *Vernonia* Schreb

**DOI:** 10.1371/journal.pone.0093149

**Published:** 2014-04-15

**Authors:** Maria Elvira Poleti Martucci, Ric C. H. De Vos, Carlos Alexandre Carollo, Leonardo Gobbo-Neto

**Affiliations:** 1 University of São Paulo (USP), School of Pharmaceutical Sciences of Ribeirão Preto, Ribeirão Preto, SP, Brazil; 2 BU Bioscience, Plant Research International,Wageningen, The Netherlands; 3 Centre for Biosystems Genomics, Wageningen, The Netherlands; 4 Netherlands Metabolomics Centre, Einsteinweg, Leiden, The Netherlands; 5 University of Mato Grosso do Sul (UFMS), Laboratory of Pharmacognosy, Campo Grande, MS, Brazil; Islamic Azad University-Mashhad Branch, Mashhad, Iran, Iran (Islamic Republic of)

## Abstract

The taxonomic classification of the genus *Vernonia* Schreb is complex and, as yet, unclear. We here report the use of untargeted metabolomics approaches, followed by multivariate analyses methods and a phytochemical characterization of ten *Vernonia* species. Metabolic fingerprints were obtained by accurate mass measurements and used to determine the phytochemical similarities and differences between species through multivariate analyses approaches. Principal component analysis based on the relative levels of 528 metabolites, indicated that the ten species could be clustered into four groups. Thereby, *V. polyanthes* was the only species with presence of flavones chrysoeriol-7*-O*-glycuronyl, acacetin-7*-O*-glycuronyl and sesquiterpenes lactones piptocarphin A and piptocarphin B, while glaucolide A was detected in both *V. brasiliana* and *V. polyanthes*, separating these species from the two other species of the *Vernonanthura* group. Species from the *Lessingianthus* group were unique in showing a positive response in the foam test, suggesting the presence of saponins, which could be confirmed by metabolite annotation. *V. rufogrisea* showed a great variety of sesquiterpene lactones, placing this species into a separate group. Species within the *Chrysolaena* group were unique in accumulating clovamide. Our results of LC-MS-based profiling combined with multivariate analyses suggest that metabolomics approaches, such as untargeted LC-MS, may be potentially used as a large-scale chemotaxonomical tool, in addition to classical morphological and cytotaxonomical approaches, in order to facilitate taxonomical classifications.

## Introduction

The tribe Vernonieae has a Pantropical distribution, being widely present in the New and Old Worlds. In Brazil the tribe is represented by around 40 genera and 450 species [1]–[4]. The genus *Vernonia* Schreb, subtribe Vernoniinae, is one of the largest groups in the Asteraceae family and includes more than 1000 species [5], [6]. In South America, there are around 350 species that mainly occur in Northern Argentina, Paraguay, Bolivia and Brazil, the later with approximately 200 species [1]–[3].

Despite the fact that the subtribe Vernoniinae is well established from a taxonomic point of view, there are several classification divergences concerning the generic limits of the *Vernonia* genus [3], [7], [8], [9], [10]. The species within this genus present a great variability in habit and morphology, leading to diverse criteria of taxonomic delimitation [11]. For example, Robinson (1999) suggested segregating several New World species into several new groups (genera), the most representative of them being *Lessingianthus*, *Chrysolaena*, *Lepidaploa* and *Vernonanthura*, thereby mainly restricting the genus *Vernonia* to those species growing in North America.

However, this reclassification of the New World species, all originally classified as *Vernonia sensu* Baker [10], into new genera has not generally been accepted, since the elevation of the different sections to generic level may be premature and does not resolve the taxonomical problem [8]. It can thus be stated that the taxonomical classification of the genus *Vernonia* is complex and needs further studies. A comprehensive phytochemical characterization of species within this genus may provide helpful chemotaxonomic information that can be used together with the classical morphological and cytotaxonomical data for a more proper and accurate classification of species within this genus [3], [8], [12]. Recent innovation in untargeted metabolomics approaches, aiming to analyze and compare samples for as many as possible of the detected compounds (both known compounds and yet unknowns) can provide a detailed insight into the differences and similarities in phytochemical composition resulting related from genetic background [13], [14].

With regard to previous phytochemical analyses of leaves from plants of the genus *Vernonia sensu* Baker, in both North and South America this genus is characterized by the presence of both flavonoid classes, such as flavones and flavonols, and sesquiterpene lactones (SLs) [1] belonging to the classes of germacranolides [15], such as glaucolides [16], hirsutinolides [17], cadinanolides [18], and guaianolides [19]. Furthermore, several saponins, for instance vernonioside D_1_, D_2_ and E, have been identified in *Vernonia amygdalina* [20], [21]. In addition, there are reports on the presence of coumarins [1] while diterpenes and alkaloids seem to be absent [5], [22].

We here report the use of untargeted metabolomics approaches, employing HPLC(DAD)-MS(ESI-QTOF), followed by multivariate analyses methods and a phytochemical characterization of ten *Vernonia* species (*sensu* Baker). For that we compare our results to the classification proposed by Robinson (1999), with the intention of evaluating if untargeted metabolomics could be employed as a chemotaxonomic tool in order to help taxonomical classifications.

## Results and Discussion

**Figure 1:**
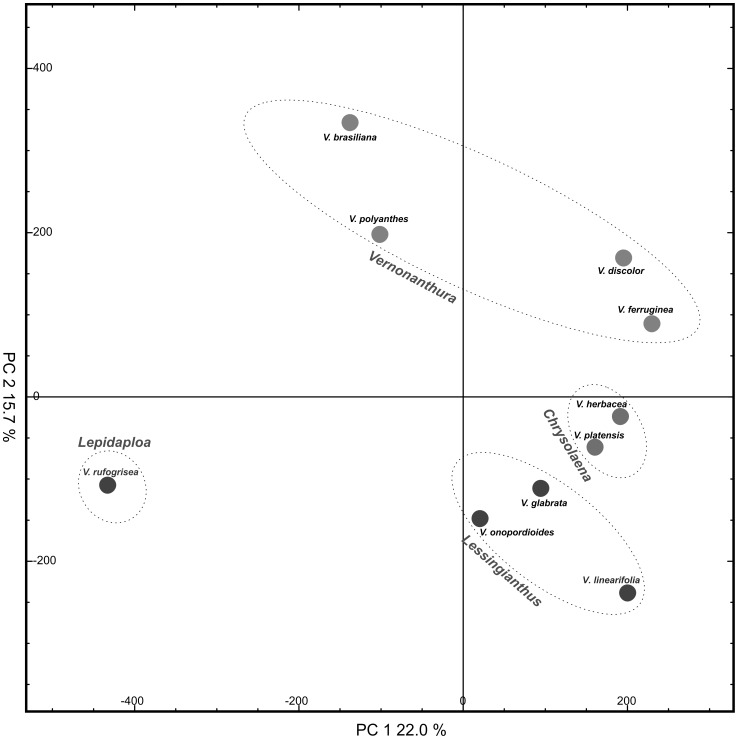
Score scatter plots of principal component analysis (PCA1 versus PCA2) of *Vernonia* species. Based on untargeted metabolic fingerprints obtained in positive ionization mode.

**Figure 2:**
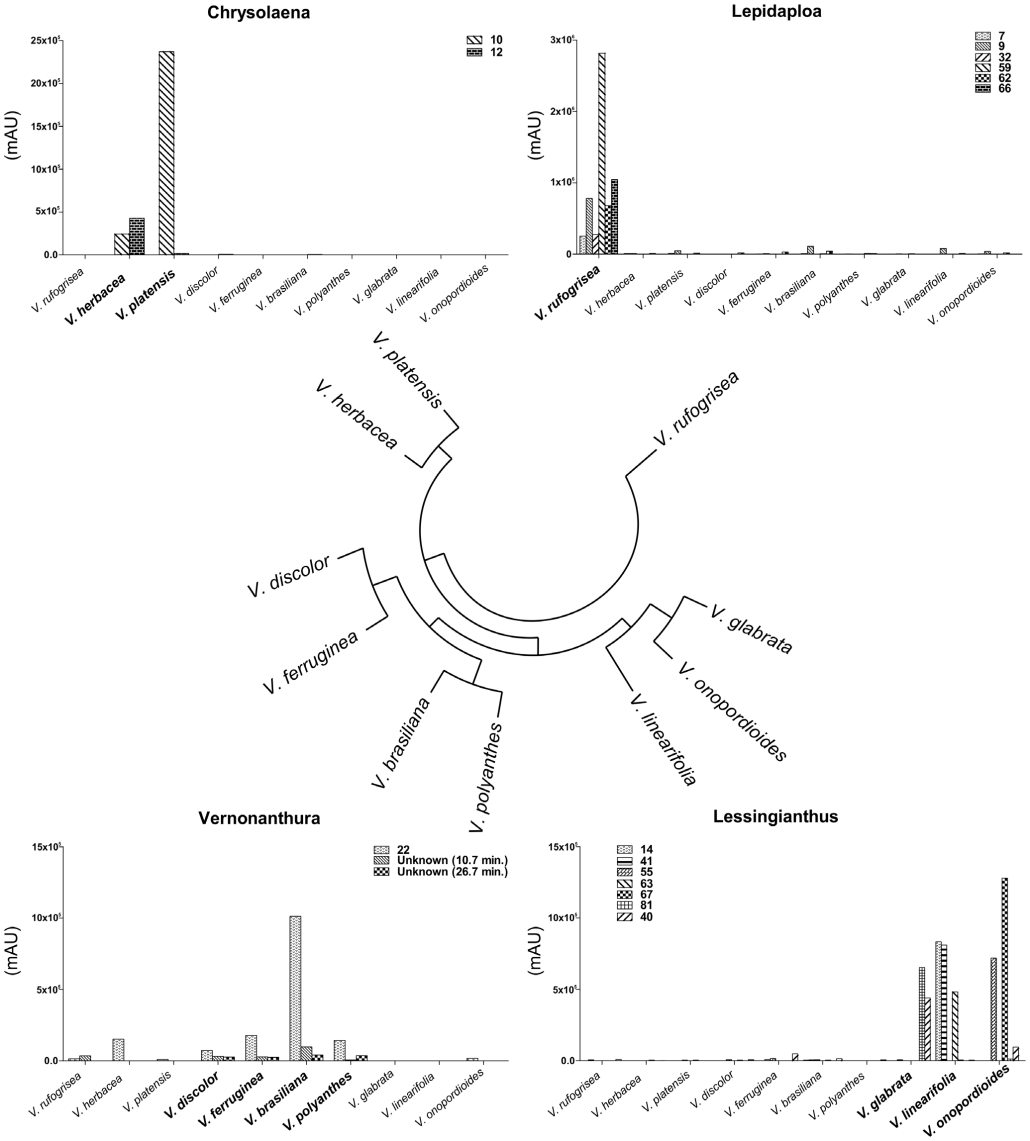
Hierarchical cluster analysis (HCA) of *Vernonia* species. Based on metabolic fingerprinting obtained in positive ionization mode and contribution of compounds to clustering. Compounds assignments are listed in Table 1.

LC-MS-based metabolic fingerprinting of crude aqueous-methanol extracts prepared from dried leaves was performed for all species, in both positive and negative electrospray ionization (ESI) modes. The data obtained in positive mode were automatically processed by MetAlign and redundant peaks removed using MSClust software, then these final reconstructed metabolite features were submitted to multivariate analysis. The resulting principal component analysis (PCA) and hierarchical cluster analysis (HCA) are shown in Fig. 1 and 2, respectively. Concomitantly, the main chromatographic peaks were identified.

For compound identification, UV spectra were used to infer the secondary metabolite class of the major chromatographic peaks, while the accurate mass obtained for the molecular ions ([M+H]^+^ and/or [M−H]^−^) were used to calculate possible molecular formula, considering a maximum deviation between observed and calculated mass of 5 ppm. The molecular formula and accurate mass were used as entry for Scifinder and Dictionary of Natural Products databases.

Product ion spectra (MS/MS) of selected precursor ions formed by collision-induced dissociation (CID) fragmentation were performed for determination/confirmation of compound annotation. The MS/MS spectra were compared with literature reports for chlorogenic acids [23], [24], flavonoids [25], [26], and sesquiterpene lactones [24]. The annotation of chromatographic peaks was performed partially based on previous phytochemical studies of *Vernonia* species. Whenever possible, retention time comparisons with authentic standards were carried out.

The metabolic profiling of plants analyzed led to the putative identification of 81 compounds, comprising chlorogenic acids, flavonoids, SLs and saponins, several of which are described here for the first time in the genus *Vernonia* Schreb. Table 1 shows the compound identities and the differentially annotated compounds accumulating in the various species analyzed. Detailed mass spectrometry information for identification of all 81 compounds is provided as Supporting Information S1.

**Table 1:** HPLC chromatographic peaks identified in species from *Vernonia* Schreb.

Peak	Compound	A	B	C	D	E	F	G	H	I	J
1	3-*O*-(*E*)-caffeoylquinic acid				X		X	X		X	
2	not identified				X						
3	5-*O*-(*E*)-caffeoylgalactaric acid									X	
4	5-*O*-(*E*)-caffeoylquinic acid	X	X	X	X	X	X	X	X	X	X
5	4-*O*-(*E*)-caffeoylquinic acid						X	X	X	X	
6	5-*p*-coumaroylquinic acid							X			
7	luteolin-6,8-di-*C*-hexoside										X
8	5-*O*-(*E*)-feruloylquinic acid							X			X
9	vicenin-2					X	X			X	X
10	clovamide								X	X	
11	quercetin-3-*O*-desoxy-hexose-*O*-hexose-*O*-pentoside						X				
12	orientin									X	X
13	quercetin-3-*O*-hexose-*O*-pentoside					X	X				
14	quercetin-3-*O*-di-hexose-*O*-pentose						X				
15	vitexin	X									X
16	kaempferol-3-*O*-hexose-*O*-desoxy-hexose-*O*-pentoside						X				
17	isovitexin										X
18	eryodictyol-glycuronyl				X				X		
19	Rutin		X	X		X	X	X	X	X	X
20	quercetin-3-*O*-dipentoside						X				
21	luteolin-7-*O*-glycuronyl				X						
22	isoquercetrin	X	X	X	X				X	X	
23	kaempferol-3-*O*-hexose-*O*-pentoside						X				
24	quercetin-3-*O*-glycuronyl					X					
25	kaempferol-3-*O*-rutinoside							X			
26	3,4-di-*O*-(*E*)-caffeoylquinic acid	X	X	X	X	X	X	X	X		
27	quercetin-3-*O*-malonyl-hexoside									X	
28	C_18_H_16_O_6_					X					
29	3,5-di-*O*-(*E*)-caffeoylquinic acid	X	X	X	X	X	X	X	X	X	X
30	eriodictyol-glycuronyl	X									
31	apigenin-7-*O*-glycuronyl	X			X						
32	hesperetin-7-*O*-rhamnoglucoside										X
33	quercetin-3-*O*-di-hexose-*O*-pentoside						X				
34	chrysoeriol-7-*O*-neohesperidoside									X	X
35	4,5-di-*O*-(*E*)-caffeoylquinic acid	X	X	X	X	X	X	X		X	X
36	kaempferol-3-*O*-malonylhexoside								X		X
37	chrysoeriol-7-*O*-glycuronyl				X						
38	acetoxy-hydroxy-methylhirsutinolide										X
39	3,4-O-(*E*)-*p*-coumaroylcaffeoylquinic acid							X			
40	3,5-*O*-(*E*)-caffeoyl-*p*-coumaroylquinic acid							X			
41	kaempferol-3-*O*-di-hexose-*O*-pentose						X				
42	8α-acetoxy-10α-hydroxy-13-*O*-methylhirsutinolide										X
43	3,4-*O*-(*E*)-caffeoylferuloylquinic acid							X			
44	3,4-*O*-(*E*)-feruloylcaffeoylquinic acid							X			
45	3,4-*O*-(*E*)-caffeoyl-*p*-coumaroylquinic acid							X			
46	isoorientin 3″-*O*-glucopyranoside						X				
47	4,5-*O*-(*E*)-caffeoyl-*p*-coumaroylquinic acid							X			
48	quercetin-3-*O*-(4″′-*O*-*trans*-caffeoyl)-α- rhamnopyranosyl-(1→6)-β-galactopyranoside							X			
49	3,4-*O*-(*E*)-caffeoyl-*p*-coumaroylquinic acid							X			
50	quercetin-3-*O*-methacrylate					X					
51	8α,13-diacetoxy-10α-hydroxyhirsutinolide										X
52	putative 1,4-epoxy-1-methoxy-8,13-diacetoxy-10-hydroxygermacra-5(*E*),										X
53	di-*O*-*p*-coumaroylquinic					X		X			
54	diacetoxy-hydroxyhirsutinolide									X	
55	kaempferol-3-*O*-hexose-*O*-caffeoyl-*O*-rhamnoside							X			
56	luteolin									X	X
57	3,4-di-*O*-(*E*)-*p*-coumaroylquinic acid							X			
58	tiliroside									X	
59	Isorhamnetin										X
60	acacetin-7-*O*-glycuronyl				X						
61	putative 8β-propioniloxy-10β-hidroxyhirsutinolide-13-*O*-acetate										X
62	kaempferol										X
63	C_40_H_66_O_15_, putative saponin						X				
64	putative 8β-acetoxy-10β-hidroxyhirsutinolide-1,13-*O*-diacetate										X
65	piptocarphin-A	X			X						
66	glaucolide B										X
67	C_46_H_74_O_16_, putative saponin							X			
68	C_40_H_66_O_14_, putative saponin						X				
69	3,7-dimethoxy-5,3′,4′-trihydroxyflavone	X									
70	C_40_H_66_O_14_, putative saponin						X				
71	C_42_H_68_O_11_, putative saponin							X			
72	C_40_H_66_O_14_, putative saponin						X				
73	C_46_H_74_O_16_, putative saponin							X			
74	glaucolide A				X						
75	piptocarphin B				X						
76	8, 8″-methylene-bisquercetin										X
77	not identified							X			
78	3′,4′-dimethoxyluteolin				X						
79	C_46_H_74_O_15_, putative saponin							X			
80	C_42_H_68_O_11_, putative saponin							X			
81	C_40_H_66_O_13_, putative saponin					X					

Species *sensu* Robinson (1999). A: *Vernonanthura brasiliana*, B: *V. discolor*, C: *V. ferruginea*, D: *V. phosphorica*, E: *Lessingianthus glabratus*, F: *L. linearifolius*, G: *L. onoporoides*, H: *Chrysolaena herbacea*, I: *C. platensis*, J: *Lepidaploa rufogrisea*. Species *sensu* Baker (1873): A: *Vernonia brasiliana*, B: *V. discolor*, C: *V. ferruginea*, D: *V. phosphorica*, E: *V. glabrata*, F: *V. linearifolius*, G: *V. onopordioides*, H: *Chrysolaena herbacea*, I: *V. platensis*, J: *V. rufogrisea*.

When comparing the metabolite profiling and multivariate analysis results with the classification proposed by Robinson (1999) for the plants from *Vernonia* genus, i.e. the segregation of these species into new genera, one can note that our results are in agreement with this classification. As proposed by Robinson (1999), *V. brasiliana*, *V. discolor*, *V. ferruginea* and *V. polyanthes* should be considered as belonging to the *Vernonanthura* group, while *V. linearifolia*, *V. glabrata* and *V. onopordioides* belong to the *Lessingianthus* group, *V. herbacea* and *V. platensis* to the *Chrysolaena* group, and *V. rufogrisea* to the *Lepidaploa* group.

SLs were only found in *V. brasiliana*, *V. platensis*, *V. polyanthes* and *V. rufogrisea*. *V. rufogrisea* was the only species accumulating 8α-acetoxy-10α-hydroxy-13-*O*-methylhirsutinolide; acetoxy-hydroxy-methylhirsutinolide; 1,4-epoxy-1-methoxy-8,13-diacetoxy-10-hydroxygermacra-5(E),7(11)-dien-6,12-olide; 8β-propioniloxy-10β-hidroxyhirsutinolide-13-*O*-acetate; 8β-acetoxy-10β-hidroxyhirsutinolide-1,13-*O*-diacetate and glaucolide B. The presence of this great and unique variety of SLs in *V. rufogrisea* is in agreement with the classification proposed by Robinson (1999), placing *V. rufogrisea* apart from the other species studied, since this species is unique within the group of *Lepidaploa* (*sensu* Robinson). This separation of *V. rufogrisea* could be clearly observed in the PCA (Fig. 1), which confirms that differentiation also occurs on metabolic level. The SL 8α,13-diacetoxy-10α-hydroxyhirsutinolide was found in both *V. rufogrisea* and *V. platensis*. *V. polyanthes* was the only species that showed accumulation of the SLs piptocarphin A and piptocarphin B, while glaucolide A was also found in *V. brasiliana*, separating these two species from two other species from *Vernonanthura* group, *V. discolor* and *V. ferruginea*. Also, HCA (Fig. 2) clearly shows that *V. discolor* and *V. ferruginea* belong to *Vernonanthura* group. In spite of PCA (Fig. 1) showing both species very close to *Chrysolaena* group, y-axis explains *Vernonanthura* group separation. The great difference observed in SLs profiles from the species studied suggests that this class may not be the best chemical markers for the genus *Vernonia* as they are being used for Asteraceae in general [15], [27], [28].

Regarding the contribution of flavonoids in the classification of some species, it should be noted that isoquercetrin was found in all species of the *Vernonanthura* group, while rutin was only found in *V. discolor* and *V. ferruginea*. On the other hand, the flavones chrysoeriol-7-*O*-glycuronyl and acacetin-7-*O*-glycuronyl were found in *V. polyanthes* only, while the flavanone hesperetin-7-*O*-rhamnoglucoside was only present in *V. rufogrisea*. In addition, it was observed that *V. linearifolia* is chemically apart from the other species of the *Lessingianthus* group, being the unique species within this group accumulating isoorientin-3″-*O*-glucupyranoside, quercetin-3-*O*-di-hexose-*O*-pentose and kaempferol-3-*O*-di-hexose-*O*-pentose. This difference can be observed in the multivariate analysis, in which *V. linearifolia* was separated from the other species of this group (Fig. 1 and 2).

Saponins appeared to contribute to the segregation of *V. linearifolia*, *V. glabrata* and *V. onopordioides* within the *Lessingianthus* group. These three species were unique in showing a positive response to the foam test, indicative of the presence of saponins, while LC-MS analysis also showed the presence of putative saponins only in the *Lessingianthus* group. Thus, saponins help to explain the differentiation of the *Lessingianthus* group from the others and could be an auxiliary tool for a rapid identification of species belonging to this group.

Finally, it is important to highlight the specific presence of clovamide, a *N*-coumaroyl-3-hydroxytyrosine [29], in both *V. herbacea* and *V. platensis*, placing these species away from the other species studied. This segregation is also in accordance with the classification proposed by Robinson (1999) of these two species into the *Chrysolaena* group.

## Conclusions

It was observed that the segregations of species based on PCA (Fig. 1) and HCA (Fig. 2) of their metabolic profiles are well in agreement with the latest classification proposed by Robinson (1999). For example, the species *V. brasiliana*, *V. discolor*, *V. ferruginea* and *V. polyanthes* clustered together, coinciding with the group *Vernonanthura*, while *V. glabrata* and *V. onopordioides* clustered according to the *Lessingianthus* group, *V. herbacea* and *V. platensis* clustered into the *Chrysolaena* group, while *V. rufogrisea* was separated corresponding to the *Lepidaploa* group.

Moreover, this study is the first comprehensive phytochemical report for the species *V. brasiliana*, *V. discolor*, *V. glabrata*, *V. linearifolia*, *V. onopordioides*, *V. herbacea* and *V. platensis*. Also some compounds were identified here for the first time in the genus *Vernonia* Schreb, such as 5-*O*-(*E*)-caffeoylgalactaric acid, clovamide, eryodictyol-glycuronyl, isoorientin 3″-*O*-glucopyranoside, quercetin-3-*O*-methacrylate, 8α,13-diacetoxy-10α-hydroxyhirsutinolide and 8,8″-methylene-bisquercetin.

Our results suggest that metabolic profiling and multivariate analysis might be a fast and more comprehensive tool for chemotaxonomic purposes than the classical and laborious phytochemical investigation. The results obtained using comprehensive metabolomics approaches, such as untargeted LC-MS, PCA and HCA, may be applied in chemotaxonomic studies with the aim to help taxonomical classifications, in a similar way as applied to filamentous fungi classification [30].

## Materials and Methods

### Plant material

The plants were collected during their flowering period in the years of 2009 and 2010 by Prof. Dr. Leonardo Gobbo Neto to minimize biological variations from harvest. The plants were identified by Prof. Dr. João Semir and Marcelo Monge Egea, Departamento de Botânica, Instituto de Biologia-UNICAMP, Brazil (Herbarium UEC), where voucher materials were deposited under the codes LG036 (*V. brasiliana* Druce, collected at São João Batista do Glória – S20°38′55.99″, W46°19′34.91″), LG042 (*V. discolor* Less, collected at Pirassununga – S22°0′28.66″, W47°16′9.52″), LG028 (*V. ferruginea* Less, collected at Espírito Santo do Pinhal – S22°9′20.94″, W 46°43′31.34″), LG025 (*V. glabrata* Less, collected at São João da Boa Vista – S22°1′56.31″, W46°47′49.88″), LG 053 (*V. herbacea* Rusby, collected at São João Batista do Glória – S20°39′8.71″, W46°19′57.66″, LG017 (*V. linearifolia* Less, collected at São José da Barra – S20°40′39.99″, W46°18′45.04″), LG014 (*V. onopordioides* Baker, collected at Delfinópolis S20°20′33.79″, W46°48′18.60″), LG019 (*V. platensis* Less, collected at Pirassununga S22°0′33.21″, W47°12′58.37″), LG026 (*V. polyanthes* Less, collected at Albertina – S22°11′23.30″, W46°35′11.48″), and LG030 (*V. rufogrisea* A.St.-Hil, collected at São João Batista do Glória – S20°38′14.62″, W46°16′27.87″). All plant material was dried at 35°C for 36 h immediately after harvesting and stored at −15°C prior preparation for analyses.

### General experimental procedures

The HPLC-UV-MS and HPLC-UV-MS/MS experiments were performed using a Shimadzu LC-20A HPLC apparatus with a diode array detector (CBM20A; Shimadzu) coupled to an ESI-QTOF mass spectrometer UltrOTOFq (Bruker Daltonics).

### HPLC-UV-MS and HPLC-UV-MS/MS analyses

Analyses by HPLC-UV-MS and HPLC-UV-MS/MS were performed using two Onyx monolithic columns (Phenomenex C 18, 100×4.6 mm) in sequence connected to a guard cartridge (Phenomenex C 18, 5.0×4.6 mm). Separation was performed at a flow rate of 1.2 ml/min and a gradient of H_2_O-HOAc (1%) (v/v) (A) and CH_3_CN-HOAc (1%) (v/v) (B) as mobile phases; the elution profile was: 0–3 min, 3% B; 3–30 min, 3–40% B; 30–35 min, 40–100% B; 35–40 min (column washing), 100% B; 40–45 min (column equilibration), 100 – 3% B. The DAD detector was set to record between 200–600 nm and chromatograms were registered at 230, 270 and 325 nm. The column effluent was split in a ratio of 3∶1 and the larger flow was conducted to the DAD detector and the lower one to the mass spectrometer. In the mass spectrometer, the column effluent was analyzed by ESI-MS separately in both positive and negative ionization modes and the mass spectra were acquired and processed using the software provided by the manufacturer. HPLC-MS total ion current (TIC) chromatograms were recorded between *m/z* 50 and 1000 and the following mass spectrometer parameters were maintained the same in all analyses: 1000 scans per second; spectrum interval, 2 s; drying gas flow, 6 ml/min; drying gas temperature, 180°C; nebulizer gas pressure, 4 bar. Retention times and precursor ions obtained by the HPLC-MS analysis were used as input for CID fragmentation in HPLC-MS/MS. N_2_ was used as drying, nebulizer and fragmentation gas.

### Sample preparation

The leaves of each single plant were dried under air circulation (35**°**C, 24 h) and powdered using an analytical knife mill. The samples were prepared using 20.0 mg of dry wt weighed in a glass vial and extracted with 3.0 ml of a solution of MeOH-H_2_O (7∶3, v/v) in an ultrasonic bath for 10 min. Finally, an aliquot of 1.0 ml was taken from the extract, filtered in a 0.45 µm PTFE membrane and 20 µl were analyzed as described in item 4.3.

### Chromatographic peak identification

Compounds were tentatively identified relied on UV spectrum and molecular formulae calculated from accurate mass measurements, both obtained from HPLC-UV-MS analyses. Such data were used to suggest secondary metabolites for each peak and were screened against the molecular formulae in the Scifinder and Dictionary of Natural Products databases. Hence, obtained informations were compared with the secondary chemistry previously reported for the *Vernonia* Schreb genus.

Online MS/MS (HPLC-UV-MS/MS) was also used for structure elucidations and to confirm the peak assignments. In addition, authentic standards of the following compounds available at our laboratories were used to confirm some identifications: luteolin, isorhamnetin, 3,7-dimethoxy-5,3′,4′-trihydroxyflavone, 3′,4′-dimethoxyluteolin, vicenin-2, vitexin, isovitexin, isoquercetrin, rutin, hesperetin-7-*O*-rhamnoglucoside, tiliroside, isoorientin-3″-*O*-glucupyranoside, quercetin-3-*O*-(4″′-*O*-*trans*-caffeoyl)-α-L-rhamnopyranosyl-(1→6)-β-D-galactopyranoside, 8α-acetoxy-10α-hydroxy-13-*O*-methylhirsutinolide, 8α,13-diacetoxy-10α-hydroxyhirsutinolide, piptocarphin A, glaucolide A and glaucolide B.

### Detection of saponins by foam test

The dried plants (500 mg) were put in a graduated cylinder with 2 ml of distilled water. The suspension was shaken for 15 s and a two cm layer of foam indicated the presence of saponins.

### Untargeted data processing and multivariate analysis

Mass signals from the raw data files were automatically extracted and aligned by MetAlign software [31], resulting in 4438 mass signals (determined as peak height) at a signal to noise ratio higher than 4000. Mass signals belonging to the same molecule, like isotopes, fragments and adducts, were subsequently re-grouped using MSClust software [32], resulting in 528 reconstructed metabolites and their relative intensity in each sample. Multivariate analysis was performed using GeneMaths XT software (version 2.11 - AppliedMaths), after 2 log transformation of metabolite signal intensities. Metabolite intensity signals (variables) were normalized by dividing the mean of each variable. HCA was performed by using Neighbor joining and Pearson's coefficient matrix.

### Ethics statement

The use of these species was allowed by CNPq at the Authorization for access of samples from the Brazilian Genetic Heritage (010091/2011-4).

## Supporting Information

Supporting Information S1 **Identification of HPLC chromatographic peaks of species from genus** ***Vernonia*** **Schreb.**
(DOCX)
