# Supplementary material for: Metabolomics as a Potential Chemotaxonomical Tool: Application in the Genus Vernonia Schreb
Source: PLoS One. 2014 Apr 15;9(4):e93149. doi: 10.1371/journal.pone.0093149 (PMC3988036; doi:10.1371/journal.pone.0093149)
Supplement: Supporting Information S1 — Identification of HPLC chromatographic peaks of species from genus Vernonia Schreb. (DOCX) [file pone.0093149.s001.docx]

**Metabolomics as chemotaxonomical tool: application in the genus *Vernonia* Schreb**

Maria Elvira PoletiMartucci^a^, Ric C.H. De Vos^b,c,d^, Carlos Alexandre Carollo^b,e^and Leonardo Gobbo-Neto^a*^

^a^*Universityof São Paulo (USP), SchoolofPharmaceuticalSciencesof Ribeirão Preto, Ribeirão Preto- SP, Brazil*

*^b^BU Bioscience, Plant Research International, Wageningen, The Netherlands*

*^c^Centre for Biosystems Genomics, Wageningen, The Netherlands*

*^d^Netherlands Metabolomics Centre, Einsteinweg, Leiden, The Netherlands*

*^e^University of Mato Grosso do Sul (UFMS), Laboratory of Pharmacognosy, Campo Grande-MS, Brazil*

**Supporting Information**

Chromatographic peaks annotation:

*Chlorogenic acids*

The chlorogenic acids were identified by comparison to compounds previously identified [1] and by identification keys [2, 3, 4]. Chlorogenic acids are constituted by a quinic acid unit esterified with a caffeic acid, showing UV maxima at ≈ 300 and at≈325 nm. Also, caffeoylquinic derivatives present *m/z* 353 [M - H]^-^(C_16_H_18_O_9_) as precursor ionduring negative ionization mode, showing a specific fragmentation pattern for each compound. Peaks 1, 4 and5 were identified as 3-*O*-(*E*)-caffeoylquinic acid, 5-*O*-(*E*)-caffeoylquinic acids and 4-*O*-(*E*)-caffeoylquinic acids, respectively. Peak 6 showed a precursor ion at *m/z* 337 (C_16_H_18_O_8_) and was identified as 5-*p*-coumaroylquinic acid, while peak 8 showed a precursor ion at *m/z* 367[M - H]^-^(C_17_H_20_O_9_) and was identified as 5-*O*-(*E*)-feruloylquinic acid [1, 2, 3, 4]. In additon, the negative ionization mode allowed identification of peak 3 as 5-*O*-(*E*)-caffeoyl-galactaric acid. This compound showed a precursor ion at *m/z* 371 [M - H]^-^(C_15_H_16_O_11_) and its MS/MS spectrum showed a product ion at *m/z* 209, formed after loss of the caffeoyl unit, which is characteristic for galactaric acid [5].

With regard to dicaffeoylquinic acids (diCQA), they showed a precursor ion at *m/z* 515 [M - H]^-^(C_25_H_24_O_12_), therefore it was possible to identifypeak26 as 3,4-di-*O*-(*E*)-caffeoylquinic acid,peak35 as 4,5-di-*O*-(*E*)-caffeoylquinic acid and peak29 as 3,5-di-*O*-(*E*)-caffeoylquinic acid [1].

Several caffeoyl-*p*-coumaroylquinic acids (C*p*CoQA) showing UV maxima at ≈ 299 and 316 nm and a precursor ion at *m/z* 499 [M - H]^-^(C_25_H_24_O_11_)in the negative ionization mode were observed. This precursor ion was selected for fragmentation in MS/MS mode, for further characterization of isomers. Therefore, peak39 showed a MS/MS spectrum of this precursor ion with product ionsat*m/z* 337, formed after loss of caffeoyl unit, *m/z* 191 and *m/z* 163 and was identified as 3,4-*O*-(*E*)-*p*-coumaroylcaffeoylquinic acid, while peak 45 a MS/MS spectrum with product ions *m/z* 337, *m/z* 173 (bp) and *m/z* 163 and was identified as 3,4-caffeoyl-*p*-coumaroylquinic acid. Finally, peak 40 showed product ions at *m/z* 353, *m/z*191 (bp), *m/z* 179, *m/z*163 and was identified as 3,5-*O*-(*E*)-caffeoyl-*p*-coumaroylquinic acid. Peak47showed MS/MS spectrum with product ions at *m/z* 337, *m/z* 191 (bp) and *m/z* 173 and was therefore identified as 4,5-*O*(*E*)-caffeoyl-*p*-coumaroylquinic acid[2, 3, 4].Peak57 showed a precursor ion at *m/z* 483 [M - H]^-^(C_25_H_24_O_10_)and its MS/MS spectrum showed product ions at*m/z* 337, produced after loss of coumaroyl unit; *m/z* 319 and *m/z* 163(bp)in the negative ionization mode, then it was identified as 3,4-di-*O*-(*E*)-*p*-coumaroylquinic acid. Peak 53 showed product ions at *m/z* 337, *m/z* 319, *m/z* 173 and *m/z* 163 (bp) in the negative ionization mode and was identified as di-*O*-*p*-coumaroylquinic acid.

Peak 43, identified as 3,4-*O*-(*E*)-caffeoylferuloylquinic acid, showed UV maximaabsorption at ≈ 299 and326 nm and a precursor ion at*m/z* 529[M - H]^-^ (C_26_H_26_O_12_) in the negative ionization mode. This precursor ion was fragmented in product ions at *m/z* 367 (bp), produced by the loss of caffeoyl unit, and *m/z* 353, produced by the loss of feruloyl unit. By the same way, the peak44 showed a precursor ion at *m/z*529 [M - H]^-^(C_26_H_26_O_12_), in the negative ionization mode and was identified as 3,4-*O*-(*E*)-feruloylcaffeoylquinic acid since it showed a similar UV maxima and product ions in the negative ionization mode of peak 43, but the product ion at *m/z* 353 was the base peak (bp) [2, 3, 4].

*Flavonoids*

*Flavonoid Aglycones*

Peak 56 was identified as luteolin after comparison of its retention time and UV spectrum with standard compounds. Also the MS/MS spectrum obtained in negative ionization mode presented the expected fragmentation patterns for luteolin and the precursor ion at *m/z* 287 [M + H]^+^ (C_15_H_10_O_6_) is in agreement [6]. In the other hand, kaempferol (peak 62) has the same molecular formula as luteolin, but the product ions formed in MS/MS spectrum in the positive ionization mode allowed distinction between them. Then, luteolin had *m/z* 153 as the bp, whereas, kaempferol had *m/z* 213 as the bp and other characteristic product ion at *m/z* 165 [7]. Also, during the MS/MS in the negative ionization mode, luteolin showed a characteristic ion at *m/z* 199 [8].

Peak 59 was identified as isorhamnetin due to precursor ions at *m/z* 315 [M - H]^-^ and *m/z* 317 [M + H]^+^ (C_16_H_12_O_7_). Still, MS/MS spectrum in the negative ionization mode showed product ions at *m/z* 300 and at *m/z* 271, which are characteristic of fragmentation patterns for this substance [6]. Finally this peak was compared with authentic standard and its identity was confirmed.

Peak 69 showed UV maxima at 268 and 355nm characteristic for flavones and it was identified as 3,7-dimethoxy-5,3',4'-trihydroxyflavone. The precursor ion at *m/z* 331 [M + H]^+^ (C_17_H_14_O_7_) and MS/MS spectrum with product ions at *m/z* 316, *m/z* 315, *m/z* 299 and *m/z* 287 were in accordance to this compound [9]. Also its identity wasconfirmed with authentic standards.

Peak 78 showed UV maxima at 268 and 346nm, characteristic for 3’,4’– dimethoxyflavones [10]. This peak showed precursor ions at *m/z* 315 [M + H]^+^ and at *m/z* 313 [M - H]^-^ (C_17_H_14_O_6_). The MS/MS spectrum in negative ionization mode showed product ions at *m/z* 298 and at *m/z* 283, formed after elimination of methyl groups and probably indicating a dimethoxyluteolin. These data led to identification of this peak as 3’,4’-dimethoxyluteolin [11], which was also compared with an authentic standard.

*C-glycosylflavonoids*

Peak 9, with UV maxima at ≈ 269 and 329 nm and precursor ions 593 [M - H]^-^ and 595 [M + H]^+^ (C_27_H_30_O_15_), was assigned as 6,8-di-*C*-*β*-glucupyranosylapigenin (vicenin-2) [7, 12, 13], which was reinforced by its fragmentation pattern. The MS/MS spectrum in the negative ionization mode showed product ions at *m/z* 575, correspondent to dehydration; *m/z* 503, *m/z* 473, *m/z* 383, *m/z* 353. This identification was confirmed by comparison with authentic standard.

UV spectrum of peak 7 (maxima at 269, 344and 258nm) is characteristic for flavones with two hydroxyl groups at the ring B. Also, this peak showeda precursor ion at *m/z* 609 [M - H]^-^ (C_27_H_30_O_16_) and its MS/MS spectrum presentedproduct ions with a characteristic fragmentation pattern of 6,8-di-C-hexosyl flavones. At the negative ionization mode, the product ion at *m/z* 399 represents the aglycone plus the residue of the sugars linked to it and therefore indicates the aglycone as trihydroxiflavone (luteolin, 286*u*). These data led to the identification of this peak as luteolin-6,8-di-*C*-hexoside [14]

Peaks 15 and 17 showed a precursor ion at *m/z* 433[M + H]^+^(C_21_H_20_O_10_)andwere identified as apigenin-8-*C*-glycoside (vitexin) and apigenin-6-*C*-glycoside (isovitexin), respectively. The main product ions during positive ionization mode were due to dehydrations, cleavage of sugar ring, and the loss of glycosidic methyl group as formaldehyde. Usually, the fragmentation of the C-6 isomers is more extensive, probably due to the formation of an additional hydrogen bond between the 2”-hydroxyl group of the sugar and 5 or 7’ hydroxyl group of the aglycone, which confers additional rigidity. In addition, the intensity ratios of these major fragments could be a way to differentiate these isomers [15]. At MS/MS in the positive ionization mode, the product ions obtained with cleavage of sugar ring have been proposed as diagnostic ions, since *m/z* 313 is the bp of apigenin-8-*C*-glycoside and *m/z* 283 is the bp of apigenin-6-*C*-glycoside. In contrast, an ion at*m/z* 361 has been only found in apigenin-6-*C*-glycoside, probably because of the additional hydrogen bond that is required for loss of the extra water [15]. Also, both peaks had their identity confirmed with authentic standards.

Peak 12 showed UV spectrum characteristic for a 3´,4´-diOH system in flavones and MS/MS spectrum of precursor ion at *m/z* 447 [M - H]^-^ (C_21_H_20_O_11_) showed product ions at *m/z* 357 and *m/z*327, which indicate the presence of mono-C-glycosides. Therefore, peak 12 was identified as luteolin-8-*C*-glycoside (orientin). It is important to note that the absence of a fragment ion at *m/z*429 is characteristic of luteolin-8-*C*-glycoside [14].

*O-glycosylflavonoids*

Peak 11 was identified as quercetin-3-*O*-deoxy-hexose-*O*-hexose-*O*-pentoside. This peak showeda precursor ion at *m/z*743 [M + H]^+^(C_32_H_38_O_20_) and its MS/MS spectrum showed product ions at *m/z* 611, *m/z* 449and *m/z* 303 (bp), which are due to a loss of pentose residue, hexose residue and deoxy-hexose unit, respectively[16].

Peak13 showed precursor ions at *m/z*595 [M - H]^-^ and at *m/z* 597[M + H]^+^(C_26_H_28_O_16_) and was identified as quercetin-3-*O*-hexose-pentoside.The MS/MS spectrum in positive ionization mode showed product ionsat *m/z* 465 and *m/z* 303, formed after loss of terminal pentose unit, and hexose unit, respectively [7, 16]. In the other hand, peak 33 showed a precursor ion at *m/z* 759 [M + H]^+^(C_32_H_38_O_21_) ant its MS/MS spectrum in positive ionization mode showed product ions at *m/z* 627, *m/z* 597 and*m/z* 303, formed after elimination of two hexose units leading to the assignment of quercetin-3-*O*-di-hexose-*O*-pentoside.

Peak 20 showed precursor ion at *m/z* 567 [M + H]^+^ (C_25_H_26_O_15_), which showed a fragmentation pattern characteristic presenting product ions due to the loss of terminal pentose unit at *m/z* 435 and an additional elimination of pentose unit at *m/z* 303 (bp). It wasidentified as quercetin-3-*O*-dipentoside [7]. Peak 22 showed a precursor ion at *m/z* 463 [M - H]^-^(C_21_H_20_O_12_)and its MS/MS spectrum presentedproduct ion at *m/z* 301 [14]. This peak was compared with authentic standard and identified as quercetin-3-*O*-glucoside (isoquercetrin).

Peak 16 showed a precursor ion at *m/z* 727 [M + H]^+^ (C_32_H_38_O_19_) and its MS/MS spectrum showed product ions at*m/z* 595, *m/z* 449 and *m/z* 287, which are characteristic of loss of terminal pentose unit, deoxy-hexose unit and hexose residue, respectively. That allowed the identification of this peak as kaempferol-3-*O*-hexose-*O*-deoxy-hexose-*O*-pentoside. Peak 23 showed precursor ions at *m/z* 581[M + H]^+^ and *m/z* 579[M - H]^-^ (C_26_H_28_O_15_). Inthe positive ionization mode this peak showed MS/MS spectrum with product ions at *m/z* 449 and at *m/z* 287. Therefore, peak 23 was identified as kaempferol-3-O-hexose-pentoside. Peak 41showed precursor ions at *m/z* 743 [M + H]^+^ and *m/z* 741[M - H]^-^ (C_32_H_38_O_20_). In the positive ionization mode, this peak showed a MS/MS spectrum with product ions at *m/z* 611 and at *m/z* 287, which are correspondent to a loss of terminal pentose unit and to an elimination of two hexose units, respectively and peak 41 was identified as kaempferol-3-*O*-di-hexose-pentoside. It is important to note thatproduct ion at *m/z* 287 showed a fragmentation pattern in agreement to kaempferol for all these peaks [7, 17].

Peak 19 showed a precursor ion at *m/z* 609 [M - H]^-^ (C_27_H_30_O_15_) in the negative ionization mode, while in positive ionization mode this peak showed precursor ion at *m/z* 611 [M + H]^+^ and its MS/MS spectrum showed product ions characteristics for rutin at *m/z* 465, due to loss of a rhamnosyl unit, and at *m/z* 303, formed after loss of hexose residue (162 *u*) or the direct loss of rutinoside residue (rhamnosyl-(α1→6)-glucose) unit. Therefore, these data and comparison with authentic standard led to identification of peak 19 as quercetin-3-*O*-rutinoside (rutin) [7].

Peak 25 showed a precursor ion at *m/z* 593[M - H]^-^(C_27_H_30_O_15_)and its MS/MS spectrum presenteda product ion at *m/z* 285 attributed to the elimination of a rutinoside residue. Also in the positive ionization mode, this peak showed precursor ions at *m/z* 595[M + H]^+^, at *m/z* 449, produced after loss of terminal rhamnosyl unit and at *m/z* 287 formed after elimination of glucose residue. Therefore, this peak was identified as kaempferol-*3-O*-rutinoside.

Peak 27 showed a precursor ion at *m/z* 551 [M + H]^+^, which produced a MS/MS spectrum with a product ion at *m/z* 303 formed after elimination of malonyl-hexose unit. Also,the MS/MS spectrum in the negative ionization mode showed an ion at *m/z* 505, formed after decarboxylation of the malonic acid unit [7, 18]. The calculated molecular formula was C_24_H_22_O_15_ and this peak was identified as quercetin-*3-O*-malonyl-hexoside. In a similar way, peak 36 was identified as kaempferol-3-*O*-malonyl-hexoside, since it showed precursor ions at *m/z* 535[M + H]^+^ and at *m/z* 533 [M - H]^-^ (C_24_H_22_O_14_). The MS/MS spectra, in positive and negative ionization modes, showed product ions at *m/z* 287 and at *m/z* 285, respectively, both produced after loss of malonyl-hexose unit.

Peak 32 showed precursor ions at *m/z* 611 [M + H]^+^ and at *m/z* 609 [M - H]^-^ (C_28_H_34_O_15_). The MS/MS spectrum in positive ionization mode showed product ions at *m/z* 449 and *m/z* 303 due to losses of rhamnosyl and glucose units, respectively [19, 20]. This peak was identified as hesperetin-7-*O*-rhamnoglucoside and its identity was confirmed through comparison with authentic standard.

Peak 34 showed UV spectrum with maxima at 266, 290 and 345 nm, that is characteristic of chrysoeriol, and precursor ions at *m/z* 609 [M + H]^+^ and *m/z* 607 [M - H]^-^ (C_28_H_32_O_15_).The MS/MS spectrum in the positive ionization mode showedproduct ions at *m/z* 463 and *m/z* 301, produced after elimination of146*u* followed by loss of 162*u*, indicating a disaccharide composed of rhamnose and glucose (neohesperidose). The nature of (1→2) interglycosidic linkage can be suggested by the evidence that ion at *m/z* 301 [(M + H) - 308]^+^ is much more abundant than ion at *m/z* 463 [(M + H) -146]^+^. Also,in the positive ionization mode, precursor ion at *m/z* 301showed product ions at *m/z* 286 and *m/z* 258 which are characteristics for chrysoeriol [21, 22]. Therefore, this peakwas identified as chrysoeriol-7-*O*-neohesperidoside.

Peak 24 showed UV spectrum characteristic for quercetin with a substituent in position 3 [10] and mass spectrum with a precursor ion at *m/z* 477 [M - H]^-^ (C_21_H_18_O_13_). This precursor ion showed MS/MS spectrum with a product ion at *m/z* 301, that is correspondent to the loss of a glycuronyl unit. Moreover, in the positive ionization mode, this peak showed MS/MS spectrum characteristic for quercetin. Product ions at *m/z* 275 would correspond to the loss of the CO group, at *m/z* 257 is formed after loss of CO_2_ and at *m/z* 229 formed after loss of both CO and CO_2_groups [23]. Therefore, peak 24 was identified as quercetin-3-*O*-glycuronyl.

Peak 21 showed UV spectrum characteristic for luteolin glycosilated at position 7, with maxima absorptions at 253, 345nm and 267nm (sh) [10]. In the negative ionization mode, this peak showed a precursor ion at *m/z* 461 [M - H]^-^ (C_21_H_18_O_12_) and its MS/MS spectrum showed a product ion at *m/z* 285, which is correspondent to luteolin and to the loss of a glycuronyl unit. In the positive ionization mode, this peak had precursor ions at m/z 463 [M + H]^+^ and at *m/z* 287. In addition, MS/MS spectrum of the later ion produced product ions at *m/z* 241, at *m/z* 161 and at *m/z* 153 (bp), characteristic to luteolin. Then, peak 21 was identified as luteolin-7-*O*-glycuronyl[24, 25].

Peak 31 showed UV spectrum with maxima absorption at 267 and 335nm, characteristic for apigenin with a substituent in position 7[10]. The mass spectrum in the negative ionization mode of this peak showed precursor ions at *m/z* 445 [M - H]^-^(C_21_H_18_O_11_) and at *m/z* 269, corresponding to the loss of a glycuronyl unit. In addition, in the positive ionization mode, were obtained precursor ions at *m/z* 447 [M + H]^+^ and at *m/z* 271, which showed a MS/MS spectrum with a product ion at *m/z* 153. Then, peak 31 was identified as apigenin-7*-O*-glycuronyl [25, 26].

Peak 37showed UV maxima at 267, 344 and 250nm (sh), indicating a substituent in position 7 of chrysoeriol [10]. In the positive ionization mode, this peak showed precursor ions at *m/z* 477 [M + H]^+^ (C_22_H_20_O_12_) and at *m/z* 301, corresponding to the loss of a glycuronyl unit. A further fragmentation of the precursor ion at *m/z* 301 resulted in a product ion at *m/z* 286, produced by the loss of methyl group [27]. Therefore, peak 37 was identified as chrysoeriol-*7-O*-glycuronyl.

Peak 60 showed UV spectrum with maxima absorptions at 265 and 330nm, characteristic for acacetin with a substituent in position 7 [10]. The mass spectrum showed precursor ions at *m/z* 461 [M + H]^+^ (C_22_H_20_O_11_) and at *m/z* 285, the latter corresponding to the aglycone and to the loss of a glycuronyl unit. Further fragmentation of the precursor ion at *m/z* 285 resulted in product ions at *m/z* 270 and at *m/z* 242, corresponding to fragmentation pattern of acacetin. These data led to the identification of acacetin-*7-O*-glycuronyl [28].

Peaks 18 and 30 showed UV maxima at 286 and 325 nm (sh) and a precursor ion at *m/z* 463 [M - H]^-^. The MS/MS spectrum of this ion showed a product ion at *m/z* 287, that is correspondent to the loss of glycuronyl unit. These peaks were identified as two positional isomers of eriodyctiol-glycuronyl (C_21_H_20_O_12_) [8].

Peak 58 showed precursor ions at *m/z* [M + H]^+^ 595 (C_30_H_26_O_13_) and at *m/z* 287 [29]. After comparison of relative retention time and fragmentation with an authentic standard compound, peak 58 was identified as kaempferol-*3-O*-(6-*p*-coumaroyl)-glycoside (tiliroside). Peak 55 had a fragmentation pattern similar to peak 58 and presented precursor ions at *m/z* 757 [M + H]^+^ and *m/z* 755 [M - H]^-^ (C_37_H_40_O_17_). The MS/MS spectrum obtained in the positive ionization mode showed product ions at*m/z* 611,produced after loss of a rhamnosyl unit, *m/z* 471, *m/z* 325, *m/z* 307, *m/z* 287 and *m/z* 163. The product ion at*m/z* 325 showed a fragment at *m/z* 163, indicating the presence of a caffeoyl unit, leading to the annotation of kaempferol-3-*O*-hexose-caffeoyl-rhamnoside.

Peak 46 showed UV spectrum with maxima absorptions at 264 and ≈ 328nm and a shoulder at 290 nm. This peak showed precursor ions at *m/z* 609[M - H]^-^and at*m/z* 611 [M + H]^+^ (C_27_H_30_O_16_). Comparison with literature [30] and with authentic standard led to its identification as isoorientin-3”-*O*-glucupyranoside.

Peak 47 showed precursor ions at *m/z* 773 [M + H]^+^ (C_36_H_36_O_19_), *m/z* 627, *m/z* 471, *m/z* 325, m/z 303 in the positive ionization mode. After comparison with authentic standard, it was identified as quercetin-3-*O*-(4″′-*O*-*trans*-caffeoyl)-α-L-rhamnopyranosyl-(1→6)-β-D-galactopyranoside. It is important to note that precursorion at *m/z* 325 formed a product ion at *m/z* 163, indicating the presence of a caffeoyl unit and the precursor ion at *m/z* 303 showed the product ions characteristics for quercetin, as was discussed above [31].

Peak 50 showed a precursor ion at *m/z* 385 [M - H]^-^  (C_19_H_14_O_9_) and the MS/MS led to a product ion at*m/z* 301due to putative methacrylate unit elimination. This product ion as well as UV maxima of this peak are both characteristic for quercetin as has been demonstrated above. Then, it was identified as putative quercetin-3-*O*-methacrylate [32].

Peak 76showed a precursor ion at *m/z* 617 [M + H]^+^ (C_31_H_20_O_14_). Its MS/MS spectrum presented product ions at *m/z* 315, putatively formed after quercetin unit elimination, and at *m/z* 303, which showed a fragmentation pattern characteristic for quercetin, as was discussed above. It was putatively identified as 4H-1-benzopyran-4-one-8,8'-methylenebis[2-(3,4-dihydroxyphenyl)-3,5,7-trihydroxy (8,8"-methylene-bisquercetin) [33].

*Sesquiterpene Lactones*

Peak 66 showed precursor ions at *m/z* 461 [M + Na]^+^, *m/z* 421 [(M + H) - H_2_O]^+^and*m/z* 439 [M + H]^+^ (C_21_H_26_O_10_). In the positive ionization mode, the MS/MS spectrum of the protonated moleculeproduced ions at *m/z* 421, *m/z* 379 (bp), *m/z* 361, *m/z* 337, *m/z* 319, *m/z* 277, *m/z* 259, *m/z* 241 and *m/z* 231, formed after consecutive losses of acetateand water units. After comparison with authentic standard, it was identified as glaucolide B [9, 34, 35]. Peak 64 showed precursor ion at *m/z* 439 [M + H]^+^ (C_21_H_26_O_10_) and the same product ions as peak 66. Comparison with peak 66 and with literature data for *Vernonia* genus led to the putative identification of this peak as 8β-acetoxy-10β-hidroxyhirsutinolide-1,13-*O*-diacetate [36].

Peak 42 showed precursor ion at *m/z* 369 [M + H]^+^ (C_18_H_24_O_8_). In the positive ionization mode, the MS/MS spectrum showed product ions at *m/z* 351, at*m/z* 309 suggesting neutral losses of water and acetate, respectively. Further loss of acetate followed by water led to *m/z* 291 (bp) and one more loss of water led to *m/z* 273. Also, this peak was compared with an authentic standard and its identity was confirmed as 8α-acetoxy-10α-hydroxy-13-*O*-methylhirsutinolide [37, 38, 39]. In a similar way, the peak 38 was identified as acetoxy-hydroxy-*O*-methylhirsutinolide since it showed precursor ions at *m/z* 369 [M + H]^+^ (C_18_H_24_O_8_) and at *m/z* 391 [M + Na]^+^. The MS/MS spectrum of this last precursor ion showed product ions at *m/z* 331 (bp) and at *m/z* 309, both suggesting losses of acetate, at *m/z* 291 indicating losses of acetate followed by water and at *m/z* 273 due to further loss of water.

Peak 51 showed precursor ions at *m/z* 397 [M + H]^+^ (C_19_H_24_O_9_), *m/z* 379 (bp), *m/z* 319, *m/z* 259 and *m/z* 213. In the positive ionization mode, the precursor ion *m/z* 397 showed MS/MS spectrum with product ions at *m/z* 379 and *m/z* 337, formed after neutral losses of water and acetate, respectively, at *m/z* 319 (bp) formed after loss of acetate unit, at *m/z* 277 formed after loss of acetate of *m/z* 337, at *m/z* 259 formed after loss of acetate of *m/z* 319, and at *m/z* 241 produced after dehydration. After comparison with authentic standard, it was identified as 8α,13-diacetoxy-10α-hydroxyhirsutinolide [37]. In a similar way, peak 54 was identified as diacetoxy-hydroxyhirsutinolide, since it showed the same fragmentation pattern as peak 51.

Peak 52 showed a precursor ion at *m/z* 411 [M + H]^+^ (C_20_H_26_O_9_) and MS/MS spectrum formed product ions at *m/z* 397, *m/z* 379 (bp), *m/z* 333, *m/z* 319, *m/z* 301, *m/z* 291, *m/z* 277, *m/z* 273, *m/z* 259, *m/z* 241 and *m/z* 217 formed after several eliminations of water and acetate units. Based on fragmentation pattern and on occurrence of this compound in the *Vernonia* genus, it was putatively identifiedas 1,4-epoxy-1-methoxy-8,13-diacetoxy-10-hydroxygermacra-5(*E*),7(11)-dien-6,12-olide[38]. Peak 61 showed the same precursor ion as peak 52 and was putatively identified as 8β-propioniloxy-10β-hydroxyhirsutinolide-13-O-acetate, since it was previously isolated in the *Vernonia* genus [36] and showed MS/MS spectrum with product ions at *m/z* 351 (bp) produced after neutral loss of acetate, at *m/z* 277, indicating losses of acetate followed by water and at *m/z* 259 formed by another loss of water.

The peak 65 showed UV maxima at 286 nm, suggesting extended conjugation, that is typical to the butadienolide moieties present in hirsutinolides. The MS spectrumshowed a precursor ion at *m/z* 423 [M + H]^+^ (C_21_H_26_O_9_) and its MS/MS spectrum presentedproduct ions at *m/z* 405 (bp), produced after dehydratation, *m/z* 345, formed after loss of acetate, *m/z* 337, formed after loss of methacrylate and *m/z* 319, formed after dehydration of *m/z* 337. Then, it was identified as piptocarphin A [40] and had its identity confirmed by comparison with an authentic standard. Peak 75 differs from peak 65 just by a tiglate group (100 *u*) loss in positive ionization mode instead of methacrylate (86 *u*). It presentedprecursor ion at *m/z* 437 [M + H]^+^(C_22_H_28_O_9_) and product ions at *m/z* 405 (bp) and *m/z* 319 formed after losses of tiglate and water units. Therefore, it was identified as piptocarphin B [40].

Peak 74 showed UV maxima at 230 nm, characteristic for glaucolides [41].This peak showed a precursor ion at *m/z* 465 [M + H]^+^(C_23_H_28_O_10_) and its MS/MS spectrum showed product ions at *m/z* 447 (bp), *m/z* 405 and *m/z* 387, produced after acetate elimination followed by dehydration, *m/z* 345, produced after losses of two acetate units and *m/z* 319 produced after losses of acetate and methacrylate units.It was identified as glaucolide A and compared with an authentic standard.

*Other Classes*

Peak 10 showed a precursor ion *m/z* 360 [M + H]^+^(C_18_H_17_NO_7_) and its MS/MS spectrum showed product ion at*m/z* 163, correspondent to loss of caffeoyl. UV maxima absorptions at 290 and ≈ 320nm confirm a caffeoyl moiety. Then, it was identified as clovamide (*N*-coumaroyl-3-hydroxytyrosine) [42, 43, 44].

Finally, peaks 28, 63, 67, 68, 69, 70, 71, 72, 73, 79, 80 and 81 were detected only in *V. glabrata*, *V. linearifolia* and *V. onopordioides*, which were the only species that showed a positive result during foam test, indicating that these species may producesaponins. These peaks also did not showed significant absorption on UV and mass spectra of all these peaks presentedconsecutive losses of sugars units leading to fragment ions corresponding to the aglycones. Thus, these peaks were putatively assigned to saponins, which are known to occur in some African *Vernonia* species [45].

Table S1 - Identification of HPLC chromatographic peaks of species from genus *Vernonia* Schreb and data taken from HPLC-UV-MS and HPLC-UV-MS/MS analyses

| Peak | *RR*t (min) | Compound | Positive Ionization | Positive Ionization | Negative Ionization | Negative Ionization | UV max (nm) |
| --- | --- | --- | --- | --- | --- | --- | --- |
|  |  |  | TIC Chromatogram Ions (*m/z*) | MS/MS | TIC Chromatogram Ions (*m/z*) | MS/MS |  |
| 1 | 4.6 | 3-*O*-(*E*)-caffeoylquinic acid | [M + H]^+^ 355.1026 bp^a^, [(M + H) - QA]^+^ 163 | 15eV: 355 → 163 bp | [M - H]^-^ 353.0879, [(M - H) - CAF^b^]^-^ 191 | 15eV: 353 →191 bp, 179 | 300, 325 |
| 2 | 4.9 | not identified | [M + H]^+^ 447.1295 bp, 409.1873, 303.0263,  205,1961 bp | 5eV: 409 → 205, 188; 5eV: 205→ 188 bp | - | - | 278 |
| 3 | 5.0 | 5-*O*-(*E*)-caffeoylgalactaric acid | [M + H]^+^ 373.0756 bp | 15eV: 373 → 163 bp | [M - H]^-^ 371.0611 bp | 15ev: 371 →209, 191 bp | 299, 327 |
| 4 | 7.5 | 5-*O*-(*E*)-caffeoylquinic acid | [M + H]^+^ 355.1015 bp, [(M + H) - QA]^+^ 163 | 15eV: 355 → 163 bp | [M - H]^-^ 353.0890 bp, [(M - H) - CAF]^-^ 191 | 15eV: 353 →191 bp, 179 | 299, 325 |
| 5 | 8.2 | 4-*O*-(*E*)-caffeoylquinic acid | [M + H]^+^ 355.1019 bp, [(M + H) - QA]^+^ 163 | 15eV: 355 → 163 bp | [M - H]^-^ 353.0888 bp, [(M - H) - CAF]^-^ 191 | 15eV: 353 →191, 179, 173 bp | 300, 325 |
| 6 | 9.4 | 5-*p*-coumaroylquinic acid | [M + H]^+^ 339.147 bp | - | [M - H]^-^ 337.0945, [(M - H) - Co^c^]^-^ 191 | 15 eV: 191 bp, 173 | 311 |
| 7 | 10.0 | luteolin-6,8-di-*C*-hexoside | [M + H]^+^ 611.1601 bp | 20eV: 611 → 593, 575, 557, 529, 527, 515, 497, 473 bp | [M - H]^-^ 609.1463 bp | 25eV: 609 → 489, 469, 399 | 258sh^e^, 269, 344 |
| 8  9 | 10.5  10.9 | 5-*O*-(*E*)-feruloylquinic acid  vicenin-2* | -  [M + H]^+^ 595.1644 bp | -  15eV: 595 → 577, 559, 541, 523, 457 bp, 427 | [M - H]^-^ 367.1048 bp, [(M - H) - FER^d^]^-^ 191  [M - H]^-^ 593.1502 bp | 15eV: 367 → 191 bp, 173  22eV: 593 → 575, 503, 473 bp, 383, 353 | 299, 324  269, 329 |
| 10 | 11.6 | clovamide | [M + H]^+^ 360.1079 bp | 10eV: 360 → 198, 163 bp | [M - H]^-^ 358.1925 bp, 222.0375 | 15eV: 358 → 222, 178, 161 | 290, 320 |
| 11 | 12.2 | quercetin-3-*O*-deoxy-hexose-*O*-hexose-*O*-pentoside | [M + H]^+^ 743.1981 bp | 5eV: 743 → 611, 597, 465, 449, 303 bp | [M - H]^-^ 741.188 bp | 27eV: 741 → 301, 300 | 254, 266sh 295sh, 350 |
| 12 | 12.4 | orientin | [M + H]^+^ 449.1067 bp | 15eV: 449 → 431, 413, 395, 383, 353, 329, 31, 299 bp | [M - H]^-^ 447.0930 bp | 15eV: 447 → 357, 327 | 255, 267, 290sh, 341 |
| 13 | 12.6 | quercetin-3-*O*-hexose-*O*-pentoside | [M + H]^+^ 597.1432, [(M + H) - pentose]^+^ 465.1025, [(M + H) - pentose-hexose]^+^ 303.0497 bp | 10eV: 597 → 465, 303 (bp)  20eV: 303 → 285, 275, 257 bp, 229, 201, 165, 153, 137 | [M - H]^-^ 595.1316 bp | 20eV: 595 → 301, 300 | 270, 290sh, 343 |
| 14 | 13.0 | quercetin-3-*O*-di-hexose-*O*-pentose | [M + H]^+^ 759.1828, [MH - hexose]^+^ 597.2467, [(M + H) - pentose - dihexose]^+^303.0287 bp | 10eV: 759 →: 693, 627, 303; 20eV: 303 → 285, 275, 257,229 bp, | [M - H]^-^ 757.1597 bp | 18eV: 757→ 595, 301, 300 | 257, 270sh, 290sh, 354 |
| 15 | 13.4 | vitexin* | [M + H]^+^ 433.1119 bp | 15eV: 433 → 415, 397, 379, 367, 351, 337, 313 bp, 295 | [M - H]^-^ 431.0983 bp | - | 268, 290sh, 328 |
| 16 | 13.5 | kaempferol-3-*O*-hexose-*O*-deoxy-hexose-*O*-pentoside | [M + H]^+^ 727.2053 bp, [M + H - pentose]^+^ 595.1638, [(M + H) - pentose - hexose - hexose]^+^ 287.0544 | 10eV: 727 → 595, 581, 287 bp, 279; 20eV: 287 → 259, 241, 213 bp, 165 | [M - H]^-^ 725.1930 bp | - | 265, 293sh, 341 |
| 17 | 13.6 | isovitexin* | [M + H]^+^ 433.1112 bp | 15eV: 433 → 415, 397, 379, 367, 361, 337, 313, 283 bp | [M - H]^-^ 431.0941 bp | - | 268, 290sh, 328 |
| 18 | 13.7 | eryodictyol-glycuronyl | [M + H]^+^ 465.0998 bp, [(M + H) - glycuronyl]^+^ 289.0685 | 15eV: 465 → 289; 10eV: 289 → 163, 153 bp | [M - H]^-^ 463.0987 bp | 15eV: 463→ 287 | 286, 325 sh |
| 19 | 13.8 | rutin* | [M + H]^+^ 611.1605 bp, [(M + H) - rhamnosyl]^+^ 465.1017, [(M + H) - rhamnosyl - glucose]^+^ 303.0480 | 10eV: 611 → 465, 303; 22eV: 303 → 285, 275, 257, 229 bp, 201, | [M - H]^-^ 609.1456 bp | 25eV: 609 → 301 | 254, 270sh, 295 sh, 352 |
|  |  |  |  | 165, 153 | | |  |
| 20 | 13.9 | quercetin-3-*O*-dipentoside | [M + H]^+^ 567.1341 bp, [M + H - pentose]^+^ 435.0919, [(M + H) - dipentose]^+^ 303.0493 | 10eV: 567 → 435, 417, 399, 303 bp, 211; 20eV: 303 → 285, 257, 229 bp, | [M - H]^-^ 465.1136 bp | - | 255, 267sh, 349 |
|  |  |  |  | 201, 165,0153 | |  |  |
| 21 | 14.0 | luteolin-7-*O*-glycuronyl | [M + H]^+^ 463.0854 bp, [(M + H) - glycuronyl]^+^ 287.0528 | 15eV: 463 → 287; 18eV: 287 → 269, 245, 153 bp | [M - H]^-^ 461.0695 bp | 15eV: 461 → 285 | 253, 267sh, 345 |
| 22 | 14.1 | isoquercetrin* | [M + H]^+^ 465.0988 bp [(M + H) - glycoside]^+^ 303.0469 | - | [M - H]^-^ 463.0865 bp | 20eV: 463 → 301 | 290, 348 |
| 23 | 14.1 | kaempferol-3-*O*-hexose-*O*-pentoside | [M + H]^+^ 581.1515 bp, [(M + H) - pentose]^+^ 449.1087, [(M + H) - pentose -hexose]^+^ 287.0545 | 10eV: 581 → 449, 287; 20eV: 287 → 259, 258, 241, 213 bp, 165, 153 | [M - H]^-^ 579.1264 bp | - | 268, 290sh, 349 |
| 24 | 14.2 | quercetin-3-*O*-glycuronyl | [M + H]^+^ 479.0817 bp [M + H - glycuronyl]^+^ 303.0499 | 10eV: 479 → 303; 30eV: 303, 285,275, 257 bp | [M - H]^-^ 477.0697 bp | 15eV: 477 → 301 | 254, 270sh, 295sh, 357 |
| 25 | 14.5 | kaempferol-3-*O*-rutinoside | [M + H]^+^ 595.0765 bp [(M + H) - rhamnosyl]^+^ 449.0589  [(M + H) - rhamnosyl - | 20eV: 595 → 287 | [M - H]^-^ 593.1491 bp | 20eV: 593 → 285 | 264, 295sh, 344 |
|  |  |  | glucose]^+^ 287.988 |  |  |  |  |
| 26 | 14.9 | 3,4-di-*O*-(*E)*-caffeoylquinic acid | [M + H]^+^ 517.1335 bp, [(M + H) - H_2_O]^+^ 499.0635,  [(M + H) - QA]^+^ 163.0899 | - | [M - H]^-^ 515.1193 bp | 15eV: 515→ 353, 335, 191, 179 bp, 173 | 299, 325 |
| 27 | 15.1 | quercetin-3-*O*-malonyl-hexoside | [M + H]^+^ 551.1017 bp | 10eV: 551 → 303; 15eV: 303 → 285, 257, 229 bp, 201, 165, 153 | [M - H]^-^ 549.0855 bp | 15eV: 549 → 505 bp, 301 | 255, 270,  295sh, 351 |
| 28 | 15.2 | C_18_H_16_O_6_ | - | - | [M - H]^-^ 327.1001 | - | - |
| 29 | 15.3 | 3,5-di-*O*-(*E*)-caffeoylquinic acid | [M + H]^+^ 517.1303 bp, [(M + H) - H_2_O]^+^ 499.0659, | - | [M - H]^-^ 515.1193 bp | 15eV: → 353 bp, 191, 179 bp | 299, 325 |
|  |  |  | [(M + H) - QA]^+^ 163.0989 |  |  |  |  |
| 30 | 15.7 | eriodictyol-glycuronyl | [M + H]^+^ 465.0990 bp, [(M + H) - glycuronyl]^+^ 289.0683 | 15eV: 465 → 289; 10eV: 289 → 271, 163, | [M - H]^-^ 463.0987 bp | 15eV: 463 → 287 | 283, 328sh |
|  |  |  |  | 153 bp | |  |  |
| 31 | 15.9 | apigenin-7-*O*-glycuronyl | [M + H]^+^ 447.0903 bp [(M + H) - glycuronyl]^+^ 271.0584 | 10eV: 447 → 271; 18eV: 271 → 253, 229, | [M - H]^-^ 445.0757 bp | 10eV: 445 → 269 | 267, 335 |
|  |  |  |  | 153 bp | |  |  |
| 32 | 16.0 | hesperetin-7-*O*-rhamnoglucoside* | [M + H]^+^ 611.1937 bp [(M + H) - glucose]^+^ 449.1401 | 10eV: 611 → 449, 345, 303 | [M - H]^-^ 609.1855 bp | - | 283, 339sh |
| 33 | 16.0 | quercetin-3-*O*-di-hexose-*O*-pentoside | [M + H]^+^ 759.1799 bp, [(M + H) - pentose]^+^ 627.1370  457.1369, [(M + H)– pentose - hexose - hexose]^+^303.0484 | 10eV: 759 → 627, 597, 325 bp, 303; 20eV: 303 → 285, 275, 257, 229 bp, 201, 165 | [M - H]^-^ 757.1602 bp | 25eV: 757 → 595, 301 | 252, 268,  300 sh,  332 |
| 34 | 16.2 | chrysoeriol-7-*O*-neohesperidoside | [M + H]^+^ 609.1795 bp | 15eV: 609 → 463, 301; 22eV : 301 → 286, | [M - H]^-^ 607.1683 bp | 15eV: 607 → 299 | 266, 290 sh, 345 |
| 35 | 16.4 | 4,5-di-*O*-(*E*)-caffeoylquinicacid | [M + H]^+^ 517.1338 bp, [(M + H) - H_2_O]^+^ 499.0877, | - | [M - H]^-^ 515.1196 bp | 20eV: 353, 191, 179, 173 bp | 299, 325 |
|  |  |  | [(M + H) - QA]^+^ 163.0543 |  |  |  |  |
| 36 | 16.7 | kaempferol-3-*O*-malonylhexoside | [M + H]^+^ 535.1069 bp, [(M + H) - malonyl - hexose]^+^ 287.0539 | 10eV: 535 → 287 | [M - H]^-^ 533.0946 bp | 15eV: 533 → 489 bp, 285 | 270, 345 |
| 37 | 16.8 | chrysoeriol-7*-O*-glycuronyl | [M + H]^+^ 477.1005 bp, [(M + H) - glycuronyl]^+^ 301.0681 | 10eV: 477 → 301; 12eV: 301 | [M - H]^-^ 475.0885 bp | 10eV: 475 → 299 | 250 sh, 267, 344 |
|  |  |  |  | → 286, 258 |  |  |  |
| 38 | 16.9 | acetoxy-hydroxy-methylhirsutinolide | [M + Na]^+^ 391.1378 bp, [M + H]^+^ 369.1547 | 10eV: 391 →331 bp, 309, 291, 273 | [M - H]^-^ 367.1422 bp | - | 286 |
| 39 | 17.0 | 3,4-O-(*E*)-*p*-coumaroylcaffeoylquinic acid | [M + H]^+^ 501.1363 bp | - | [M - H]^-^ 499.1243 bp | 15eV: 499 → 337, 191, 173, 163 bp | 299, 316 |
| 40 | 17.1 | 3,5-*O*-(*E*)-caffeoyl-*p*-coumaroylquinic acid | - | - | [M - H]^-^ 499.1243 bp | 15eV: 499 → 353, 337, 191 bp, 179, 163 | 299, 320 |
| 41 | 17.2 | kaempferol-3-*O*-di-hexose-*O*-pentose | [M + H]^+^ 743.1794 bp, [(M + H) - pentose]^+^ 611.1444, 457.1357, 287.0570 | 10eV: 743 → 611, 457, 325 bp, 295, 287, 163; | [M - H]^-^ 741.1682 bp | 27eV: 741 → 285 | 266, 300sh, 328 |
|  |  |  |  | 20eV: 287 → 259,  241, 213 bp | |  |  |
| 42 | 17.4 | 8α-acetoxy-10α-hydroxy-13-*O*-methylhirsutinolide* | [M + Na]^+^ 391.1378 bp, [M + H]^+^ 369.1547, 291 | 10eV: 391 → 331 bp, 309, 291, 273, 259, 241, 213; 5eV: 369 | [M - H]^-^ 367.1422 bp | - | 286 |
|  |  |  |  | → 351, 309, 291 bp, 277, 273, 259, 241, 231, 217, 215 |  |  |  |
| 43 | 17.5 | 3,4-*O*-(*E*)-caffeoylferuloylquinic acid | [M + H]^+^ 531.1335 bp | - | [M - H]^-^ 529.1371 bp | 15eV: 529 → 367 bp, 353, 191 | 299, 326 |
| 44 | 17.7 | 3,4-*O*-(*E*)-feruloylcaffeoylquinic acid | [M + H]^+^ 531.1335 bp | - | [M - H]^-^ 529.1371 bp | 15eV: 529 → 367, 353 bp, | 299, 325 |
| 45 | 18.0 | 3,4-*O*-(*E*)-caffeoyl-*p*-coumaroylquinic acid | [M + H]^+^ 501.1363 bp | - | [M - H]^-^ 499.1250 bp | 15eV: 499 → 361, 337bp, 173bp, 163 | 295, 315 |
| 46 | 18.1 | isoorientin 3”-*O*-glucopyranoside* | [M + H]^+^ 611.1421 bp, 501.1368, 325.1268, 303.1468 | 10eV: 611 → 325, 287 bp, 163; 20eV: 287 → 259, 241 213 bp | [M - H]^-^ 609.1264 bp | 15eV: 609 → 323, 285 | 264, 290sh, 328 |
|  |  |  |  | 165, 153 | |  |  |
| 47 | 18.1 | 4,5-*O*-(*E*)-caffeoyl-*p*-coumaroylquinic acid | [M + H]^+^ 501.1363 bp | - | [M - H]^-^ 499.1250 bp | 15eV: 499 → 361, 337, 191 bp 173 | 295, 315 |
| 48 | 18.2 | quercetin-3-*O*-(4″′-*O*-*trans*-caffeoyl)-α- rhamnopyranosyl-(1 → 6)-β-galactopyranoside* | [M + H]^+^ 773.2581bp, 627.1347, 471.2612, 325.1338 | 20eV: 773 → 627, 471, 325, 307, 303, 289, 163; 10eV: 627 → 325, 307, 303, 289; 10eV: 471 → 325, 163  30eV: 303→ 285, 275, 257 bp, 229, 201, 165 | [M - H]^-^ 771.1759 bp, 469.1523, 385.1347, 301.1223 | 15eV: 771 → 301 | 250, 268sh, 300, 330 |
| 49 | 18.4 | 3,4-*O*-(*E*)-caffeoyl-*p*-coumaroylquinic acid | [M + H]^+^ 501.1363 bp | - | [M - H]^-^ 499.1243 bp | 15eV: 499 → 337 bp, 191, 173 bp, 163 | 299, 316 |
| 50 | 18.5 | quercetin-3-*O*-methacrylate | - | - | [M - H]^-^ 385.0894 bp | 15eV: 385 → 301 | 270, 295sh, 332 |
| 51 | 18.6 | 8α,13-diacetoxy-10α-hydroxyhirsutinolide* | [M + H]^+^ 397.1483, 379 bp, 319.0877, 259.1344, 213.8766 | 5eV: 397 → 379, 357, 337, 319bp, 301, 277, 259, 241, 231, 217, | [M - H]^-^ 395.1368 bp | - | 286 |
|  |  |  |  | 213, 199 |  |  |  |
| 52 | 18.8 | putative 1,4-epoxy-1-methoxy-8,13-diacetoxy-10-hydroxygermacra-5(*E*), 7(11)-dien-6,12-olide | [M + H]^+^ 411.1627, 393.8976, 333.3457 bp, 301.1322, 273.7764, 199.0989 | 5eV: 411 → 397, 379 bp, 333, 319, 301, 291, 277, 273, 259, 241, 217, 213 | [M - H]^-^ 409.1563 bp | - | 286 |
| 53 | 18.9 | di-*O*-*p*-coumaroylquinic acid | [M + H]^+^ 485.1345 bp | - | [M - H]^-^ 483.1343 bp | 15eV: 483 → 337, 319, 173, 163 bp | 300, 312 |
| 54 | 19.0 | diacetoxy-hydroxyhirsutinolide | [M + H]^+^ 397.1485 bp | 10eV: 397 → 379, 345, 337, 319 bp, 277, 259, 241, 213 | [M - H]^-^ 395.1311 bp | - | 286 |
| 55 | 19.4 | kaempferol-3-*O*-hexose-*O*-caffeoyl-*O*-rhamnoside | [M + H]^+^ 757.1988 bp | 20eV: 757 → 611, 471, 325, 307, 287, 163; | [M - H]^-^ 755.1835 bp | 15eV: 755 → 624, 469, 285 bp | 266, 300, 327 |
|  |  |  |  | 10eV: 471 → 325, 163; 30eV: 287 → 259, 258, 241, 213, 163, 153 bp |  |  |  |
| 56 | 19.6 | luteolin* | [M + H]^+^ 287.0543 bp | 30eV: 287 → 269, 259, 258, 241, 213, 153 bp | [M - H]^-^ 285.0395 bp | 20eV: 285 → 241, 217, 201, 199, 184, 175 | 252, 265sh, 295sh, 353 |
| 57 | 20.1 | 3,4-di-*O*-(*E*)-*p*-coumaroylquinic acid | [M + H]^+^ 485.1345 bp | - | [M - H]^-^ 483.1343 bp | 15eV: 483 → 337, 163bp | 300, 312 |
| 58 | 20.4 | tiliroside* | [M + H]^+^ 595.1444 bp | 8eV: 595 → 309, 287, 165, 147 | [M - H]^-^ 593.1251 bp | 20eV: 593 → 285 | 265, 295sh, |
|  |  |  |  |  |  |  | 355sh |
| 59 | 20.5 | isorhamnetin* | [M + H]^+^ 317.0669 bp | 30eV: 317 → 302, 301 bp, 285, 274, 273, 257,  245, 228, 217 | [M- H]^-^ 315.0536 bp | 20eV: 315 → 300 bp, 271, 255, 243, 227, 214 | 255, 265sh, 290sh, 354 |
| 60 | 20.9 | acacetin-7-*O*-glycuronyl | [M + H]^+^ 461.1033 bp, [MH - glycuronyl]^+^ 285.0730 | 10eV: 461 → 285; 27eV: 285 → 267, 253, 239 | [M - H]^-^ 459.0923 bp | 5eV: 459 → 283 | 265, 330 |
| 61 | 22.2 | putative 8β-propioniloxy-10β-hidroxyhirsutinolide-13-*O*-acetate | [M + H]^+^ 411.1657 bp | 5eV: 411 → 351 bp, 277, 259, 241, 217, 215 | - | - | 285 |
| 62 | 22.7 | kaempferol | [M + H]^+^ 287.0541 bp | 25eV: 287 → 258, 231, 213 bp, 165, 153 | [M - H]^-^ 285.0432 bp | - | 266, 289, 363 |
| 63 | 23.6 | C_40_H_66_O_15_, putative saponin | M + H]^+^ 787.4457 bp,  [(M + H) - hexose]^+^ 625.3816, 589.3726,413.2491 | - | [M - H]^-^785.4165 | - | - |
| 64 | 23.7 | putative 8β-acetoxy-10β-hidroxyhirsutinolide-1,13-*O*-diacetate | [M + H]^+^ 439.1577, 421.1469 bp | 5eV: 439 → 421, 379 bp, 361, 337, 319, 277, 259, 241, 231, 213 | - | - | 287 |
| 65 | 24.5 | piptocarphin A* | [M + H]^+^ 423.1627, [M +Na]^+^ 445.1414, [M + K]^+^ | 5eV: 423 → 405, 345, 319 bp, 301, | - | - | 286 |
|  |  |  | 461.1657, [(M + H) - H_2_O]^+^ 405.1532 bp, [(M + H) - H_2_O - C_2_H_4_O_2_]^+^ 345.1269, [(M + H) - C_4_H_6_O_2_]^+^  337.1236, [(M + H) - C_4_H_6_O_2_ - H_2_O]^+^ 319.1144 | 277, 259, 241, 231  213; 5eV: 405 → 345,  319, 301, 277, 259,  241, 231, 213, 199,  189, 173 | |  |  |
| 66 | 24.8 | glaucolide B* | [M + H]^+^ 439.1577, [M +Na]^+^ 461.1380, [(M + H) - H_2_O]^+^ 421.1469 bp, | 5eV: 439 → 421, 379 bp, 361, 337, 319, 277, 259, 241, 231,213, 199, 171 | - | - | 230, 287 |
| 67 | 24.9 | C_46_H_74_O_16,_ putative saponin | [(601.4012) - rhamnosyl]^+^  455.3492 bp | - | 941.5122, 530.2760 bp | - | - |
| 68 | 25.9 | C_40_H_66_O_14_, putative saponin | [M + H]+ 771.4416, [(M +  H) - hexose]^+^609.3991,329.2508 bp | - | 829.4649 | - | - |
| 69 | 27.0 | 3,7-dimethoxy-5,3',4'-trihydroxyflavone* | [M + H]^+^ 331.0802 bp | 15eV: 331 → 316, 287, 25eV: | [M - H]^-^ 769.4260 | - | 268, 355 |
|  |  |  |  | 331 → 315, 288, 287,273, 245 |  |  |  |
| 70 | 27.4 | C_40_H_66_O_14_, putative saponin | [M + H]^+^ 771.4473, [(M + H) - hexose]^+^609.3981, 411.3250bp | - | [M - H]^-^ 769.4404 | - | - |
| 71 | 27.7 | C_42_H_68_O_11,_putative saponin | [M + H]+ 781.4303, [(M + H) - rhamnosyl]^+^ 635.4167, 453.3356 bp | - | 839.4977 bp  [M - H]^-^ 779.4150 | - | - |
| 72 | 28.0 | C_40_H_66_O_14_, putative saponin | [M + H]^+^ 771.4531, [(M + H) - hexose]^+^609.3987, 447.3403 | - | 829.4657 bp, [M - H]^-^ 769.4406 | - | - |
| 73 | 28.1 | C_46_H_74_O_16,_ putative saponin | [(601.4180) - rhamnosyl]^+^455.3512 bp | - | 941.5119 bp, 530.2750 | - | - |
| 74 | 28.2 | glaucolide A* | [M + H]^+^ 465.1759, [M +Na]^+^ 487.1525, [M + K]^+^ 503.1232, [(M + H) - H_2_O]^+^ 447.1650 bp, 405.1506, [(M + H) - H_2_O -C_2_H_4_O_2_]^+^ 387.1400, [(M + H) - 2C_2_H_4_O_2_]^+^ 345.1337, [(M + H) - C_2_H_4_O_2_ - C_4_H_6_O_2_]^+^ 319.1141 | 5eV: 465 → 447 bp, 405, 387, 363, 345, 319, 281, 259, 241, 213, 173 | - | - | 230 |
| 75 | 28.5 | piptocarphin B | [M + H]^+^ 437.1789, [M + Na]^+^ 459.1614, 405.1532 bp, [(M + H) - C_5_H_8_O_2_ - H_2_O]^+^ 319.1149, [(M + H) - C_5_H_8_O_2_-C_2_H_4_O_2_ - H_2_O]^+^ 259.0946 | 5eV: 437 → 405, 345, 319 bp, 277, 259, 241; 5eV: 405 → 361, 345, 319, 277, 259, 241, 213, 189 | - | - | 285 |
| 76 | 29.0 | 8, 8"-methylene-bisquercetin | [M + H]^+^ 617.0873 bp | 15eV: 617 → 327, 317, 315, 303 bp, 25eV: 617 → 327, 317, 315, 303, 302, | [M - H]^-^ 615.0782 bp, 299.0184 | 15eV: 615 → 299 | 268, 302, 350 |
|  |  |  |  | 301; 15eV: 303 |  |  |  |
|  |  |  |  | → 285 bp, 201, 165 |  |  |  |
| 77 | 29.1 | not identified | - | - | 839.4807 bp, [M - H]^-^ 779.4596 | - | - |
| 78 | 29.8 | 3’,4’-dimethoxyluteolin* | [M + H]^+^ 315.0832 bp | 18eV: 315 → 300, 272, 257, 243, 215, 169; 35eV: 315 → 300, 272, 257 | [M - H]^-^ 313.0879 | 15eV: 313 → 298, 283, 255 | 268, 346 |
| 79 | 31.5 | C_46_H_74_O_15_, putative saponin | [(585.4011) - rhamnosyl]^+^ 439.3554 bp | - | 925.5175 bp | - | - |
| 80 | 32.3 | C_42_H_68_O_11,_putative saponin | [M + Na]^+^ 803.4638, 439.3556 bp | - | 839.4802 bp, [M - H]^-^ 779.4403 | - | - |
| 81 | 32.9 | C_40_H_66_O_13,_ putative saponin | [M + Na]^+^ 777.4424, , 569.3790 bp, 407.3590 | - | [M - H]^-^ 753.4689 | - | - |

^a^bp – base peak; ^b^CAF – caffeoyl; ^c^Co – coumaroyl; ^d^FER – feruloyl; ^e^sh – shoulder

*standard compounds used for confirmation of compounds

**References**

1. Gobbo-Neto L, Lopes NP (2008a). Online identification of chlorogenic acids, sesquiterpene lactones, and flavonoids in the Brazilian arnica *Lychnophoraericoides*Mart. (Asteraceae) leaves by HPLC-DAD-MS and HPLC-DAD-MS/MS and a validated HPLC-DAD method for their simultaneous analysis. J Agric Food Chem 56: 1193-1204.
2. Clifford MN, Johnston KL, Knight S, Kuhnert N (2003) Hierarchical scheme for LC-MS^n^identiﬁcation of chlorogenic acids. J Agric Food Chem 51: 2900-2911.
3. Clifford MN, Knight S, Kuhnert N (2005) Discriminating between the six isomers of dicaffeoylquinic acid by LC-MS^n^. J Agric Food Chem 53: 3821-3832.
4. Clifford MN, Knight S, Surucu B, Kuhnert N (2006) Characterization by LC-MS^n^ of four new classes of chlorogenic acids in green coffee beans: Dimethoxycinnamoylquinic acids, diferuloylquinic acids, caffeoyl-dimethoxycinnamoylquinic acids, and feruloyl-dimethoxycinnamoylquinic acids. J Agric Food Chem 54: 1957-1969.
5. Ismail IS, Ito H, Yoshida T (2007) An ichthyotoxicprocyanidin oligomer and four new hydroxyacid conjugates from the leaves of *Sandoricumkoetjape*. ACGC. Chem Res Commun21: 13-19.
6. Santos SAO, Freire CSA, Domingues MRM, Silvestre AJD, Pascoal Neto C (2011) Characterization of phenolic components in polar extracts of *Eucalyptus globules* Labill.Bark by high-performance liquid chromatography mass spectrometry. J Agric Food Chem 59: 9386-9393.
7. Cuyckens F, Claeys M (2004) Mass spectrometry in the structural analysis of ﬂavonoids. J Mass Spectrom 39: 1-15.
8. Sánchez-Rabaneda F, Jauregui O, Lamuela-Raventos RM, Bastida J, Viladomat F, et al. (2003) Identiﬁcation of phenolic compounds in artichoke waste by high-performance liquid chromatography–tandem mass spectrometry. J Chromatogr A. 1008: 57-72.
9. Alarcon MABV, Lopes JC, HerzW (1990) Glaucolide B, a molluscicidalsesquiterpene lactone, and other constituents of *Vernoniaeremophila.* Planta Med 56: 271-273.
10. Markham KR (1982) Techniques of Flavonoid Identification. Londos: Academic Press.
11. Kuwabara H, Kyoko M, Hideaki O, Ryoji K, Kasuo Y (2003) Tricin from a *Malagasy connaraceous* plant with potent antihistaminic activity. J Nat Prod 66: 1273-1275.
12. Gobbo-Neto L, Gates PJ, Lopes, NP (2008b) Negative ion ‘chip-based’ nanospray tandem mass spectrometry for the analysis of flavonoids in glandular trichomes of *Lychnophoraericoides* Mart. (Asteraceae). Rapid Commun Mass Spectrom 22: 3802-3808.
13. Iswaldi I, Arráez-Román D, Rodríguez-Medina I, Beltrán-Debón R, Joven, J, et al.(2011) Identification of phenolic compounds in aqueous and ethanolic rooibos extracts (*Aspalathuslinearis*) by HPLC-ESI-MS (TOF/IT). Anal Bioanal Chem 400: 3643 – 3654.
14. Breiter T, Laue C, Kressel G, Gröll S, Engelhardt UH, et al. (2011) Bioavailability and antioxidant potential of rooibos ﬂavonoids in humans following the consumption of different rooibos formulations. Food Chem128: 338-347.
15. Abad-García B, Garmón-Lobato S, Berrueta LA, Gallo B, Vicente F (2008) New features on the fragmentation and differentiation of C-glycosidic ﬂavone isomers by positive electrospray ionization and triple quadrupole mass spectrometry. Rapid Commun Mass Spectrom 22: 1834–1842.
16. Gil-Izquierdo A, Mellenthin A (2001) Identification and quantitation of flavonols in rowanberry (*Sorbusaucuparia* L.) juice. Eur Food Res Technol213: 12–17.
17. Vallverdú-Queralt A, Jáuregui O, Medina-Remón A, Andrés-Lacueva C, Lamuela-Raventós RM (2010) Improved characterization of tomato polyphenols using liquid chromatography/electrospray ionization linear ion trap quadrupoleOrbitrap mass spectrometry and liquid chromatography/electrospray ionization tandem mass spectrometry. Rapid Commun Mass Spectrom 24: 2986-2992.
18. Kachlicki P, Einhorn J, Muth D, Kerhoas L, Stobiecki M (2008) Evaluation of glycosylation and malonylation patterns in ﬂavonoid glycosides during LC/MS/MS metabolite profiling. J Mass Spectrom 43: 572–586.
19. Kim HG, Kim GS, Lee JH, Park S, Jeong WY, et al.(2011) Determination of the change of ﬂavonoid components as the defence materials of *Citrus unshiu* Marc. fruit peel against *Penicilliumdigitatum* by liquid chromatography coupled with tandem mass spectrometry. Food Chem 128: 49-54.
20. Martín, T, Rubio B, Villaescusa L, Fernández L, Díaz AM (1999) Polyphenolic compounds from pericarps of *MyrtusCommunis.* Pharm Biol 37: 28-31.
21. Gattuso G, Caristi C, Gargiulli C, Bellocco E, Toscano G, et al.(2006) Flavonoid Glycosides in Bergamot Juice (*Citrus bergamia*Risso). J Agric Food Chem 54: 3929-3935.
22. Qi LW, Chen CY, Li P (2009) Structural characterization and identiﬁcation of iridoid glycosides, saponins, phenolic acids and ﬂavonoids in *FlosLoniceraeJaponicae* by a fast liquid chromatography method with diode-array detection and time-of-ﬂight mass spectrometry. Rapid Commun Mass Spectrom 23: 3227-3242.
23. Dueñas M, Mingo-Chornet H, Pérez-Alonso JJ, Di Paola-Naranjo R, González-Paramás AM, et al. (2008) Preparation of quercetinglucuronides and characterization by HPLC–DAD–ESI/MS. Eur Food Res Techno l227: 1069-1076.
24. Nagy TO, Solar S, Sontag G, Koenig J (2011) Identiﬁcation of phenolic components in dried spices and inﬂuence of irradiation. Food Chem 128: 530-534.
25. Surowiec I, Szostek B, Trojanowicz M (2007) HPLC-MS of anthraquinoids, flavonoids, and their degradation products in analysis of natural dyes in archeological objects. J Sep Sci 30: 2070-2079.
26. Petroviciu I, Albu F, Medvedovici A (2010) LC/MS and LC/MS/MS based protocol for identiﬁcation of dyes in historic textiles. MicrochemJ 95: 247-254.
27. El-Hela AA, Al-Amier HA, Ibrahim TA (2010) Comparative study of the ﬂavonoids of some Verbena species cultivated in Egypt by using high-performance liquid chromatography coupled with ultraviolet spectroscopy and atmospheric pressure chemical ionization mass spectrometry. J Chromatogr A 1217: 6388-6393.
28. Shi P, Zhang Y, Qu H, Fan X (2011) Systematic characterisation of secondary metabolites from *Ixerissonchifolia* by the combined use of HPLC-TOFMS and HPLC-ITMS. Phytochem Anal 22: 66-73.
29. Aguirre-Hernández E, Gonzáles-Trujano MaE, Martínez AL, Moreno J, Kite G, et al. (2010) HPLC/MS analysis and anxiolytic-like effect of quercetin and kaempferol flavonoids from *Tiliaamericana* var. *Mexicana*. J Ethnopharmacol 127: 91-97.
30. Deng X, Gao G, Zheng S, Li F (2008) Qualitative and quantitative analysis of ﬂavonoids in the leaves of *Isatisindigatica* Fort. by ultra-performance liquid chromatography with PDA and electrospray ionization tandem mass spectrometry detection. J Pharm Biomedical Anal 48: 562-567.
31. Li J, Jiang H, Shi R (2009) A new acylated quercetin glycoside from the leaves of *Stevia rebaudiana*Bertoni. Nat Prod Res 23: 1378-1383.
32. Montenegro L, Carbone C, Maniscalco C, Lambusta D, Nicolosi G, et al. (2007) In vitro evaluation of quercetin-3-*O*-acyl esters as topical prodrugs. Int J Pharm 336: 257262.
33. Li N, Zhang C, Zhang MA (2008) New biflavonoid from *Senecioargunensis*Turcz. ZhongguoYaokeDaxueXuebao39: 20-22.
34. Jakupovic J, Banerjee S, Castro V, Bohlmann F, Schuster, A, et al. (1986a) Poskeanolide, a seco-germacranolide and other sesquiterpene lactones from*Vernonia* species. Phytochem25: 1359-1364.
35. Padolina WG, Yoshioka H, Nakatani N, Mabry J, Monti SA, et al. (1974) Glaucolide-A and B, nemgermacranolide-type esquiterpene lactones from *Vernonia* (Compositae). Tetrahedron 30: 1161-1170.
36. Bohlmann F, Mhanta PK, Dutta LN (1979) Weitere hirsutinolide aus *Vernonia*-Arten*. Phytochem18: 289-291.
37. Appezzato-da-Glória B, Da Costa FB, Da Silva VC, Gobbo-Neto L, Rehder, VLG, et al. (2012) Glandular trichomes on aerial and underground organs in *Chrysolaena*species (Vernonieae-Asteraceae): Structure, ultrastructure and chemical composition. Flora 207: 878-887.
38. Bardón A, Montanaro S, Catalán CAN, Diaz JG, Herz W (1993) Piptocarphols and other constituents of *Chrysolaena Verbascifolia* and *Lessingianthus Rubricaulis*. Phytochem 34: 253-259.
39. Jakupovic J, Schmeda-Hirschmann G, Schuster A, Zdero C, Bohlmann F, et al. (1986b) Hirsutinolides, glaucolides and sesquiterpene lactone from *Vernonia* species. Phytochem 25: 145-158.
40. Cowall PL, Cassady JM, Chang C, Kozlowski JF (1981) Isolation and structure determination of piptocarphins A-F, cytotoxic germacranolide lactones from *Piptocarphachontalensis*. J Org Chem 46: 1114-1120.
41. Bardón A, Montanaro S, Catalán CAN, Gutiérrez AB, Herz W (1990) Glaucolides and related sesquiterpene lactones from *Vernoniaincana*. Phytochem 29: 313-315.
42. Polasek J, Queiroz EF, Hostettmann K (2007) On-line identiﬁcation of phenolic compounds of *Trifolium* species using HPLC-UV-MS and post-column UV-derivatisation. Phytochem Anal 18: 13–23.
43. Stark T, Hofmann T (2005) Isolation, structure determination, synthesis, and sensory activity of N-Phenylpropenoyl-L-aminoacids from Cocoa (*Theobroma cacao*). J Agric Food Chem 53: 5419-5428.
44. Pereira-Caro G, Borges G, Nagai C, Jackson MC, Yokota T, et al. (2013) Profiles of phenolic compounds and purine alkaloids during the development of seeds *Theobroma cacao* cv. Trinitario. J Agric Food Chem 61: 427-434.
45. Schmittmann T, Rotscheidt K, Breitmaier E (1994) Three new steroid saponins from *Vernonia amygdalina* (Compositae). Journal fuer Praktische Chemie/Chemiker-Zeitung 336: 225-232.
